# Expression of Adrenoceptor Subtypes in Preterm Piglet Heart Is Different to Term Heart

**DOI:** 10.1371/journal.pone.0092167

**Published:** 2014-03-26

**Authors:** Min Young Kim, Angela M. Finch, Eugenie R. Lumbers, Amanda C. Boyce, Karen J. Gibson, Yvonne A. Eiby, Barbara E. Lingwood

**Affiliations:** 1 The University of Queensland, UQ Centre for Clinical Research, Brisbane, Australia; 2 Department of Pharmacology, School of Medical Sciences, The University of New South Wales, Sydney, Australia; 3 Department of Physiology, School of Medical Sciences, The University of New South Wales, Sydney, Australia; 4 School of Biomedical Sciences and Pharmacy, University of Newcastle, Newcastle, Australia; Cincinnati Children’s Medical Center, United States of America

## Abstract

Preterm delivery increases the risk of inadequate systemic blood flow and hypotension, and many preterm infants fail to respond to conventional inotrope treatments. If the profile of cardiac adrenoceptor subtypes in the preterm neonate is different to that at term this may contribute to these clinical problems. This study measured mRNA expression of β_1_, β_2_, α_1A_, α_2A_ and α_2B_-adrenoceptor subtypes by real time PCR in term (113d), preterm (91d) and preterm piglets (91d) exposed to maternal glucocorticoid treatment. Abundance of β-adrenoceptor binding sites in the left ventricle was measured using saturation binding assays. Relative abundance of β_1_-adrenoceptor mRNA in untreated preterm hearts was ∼50% of term abundance in both left and right ventricles (*P*<0.001). Trends in receptor binding site density measurements supported this observation (*P* = 0.07). Glucocorticoid exposure increased β_1_-adrenoceptor mRNA levels in the right ventricle of preterm hearts (*P* = 0.008) but did not alter expression in the left ventricle (*P*>0.1). Relative abundance of α_1A_-adrenoceptor mRNA was the same in preterm and term piglet hearts (*P* = >0.1) but was reduced by maternal glucocorticoid treatment (*P*<0.01); α_2A_-adrenoceptor mRNA abundance was higher in untreated and glucocorticoid exposed preterm piglet hearts than in term piglets (*P*<0.001). There was no difference between male and female piglets in mRNA abundance of any of the genes studied. In conclusion, there is reduced mRNA abundance of β_1_-adrenoceptors in the preterm pig heart. If this lower expression of β-adrenoceptors occurs in human preterm infants, it could explain their poor cardiovascular function and their frequent failure to respond to commonly used inotropes.

## Introduction

Following preterm birth, many neonates exhibit low systemic blood flow (SBF) and/or hypotension [1], and this is associated with an increased risk of infant morbidity and mortality [2], [3], [4]. Moreover, a significant proportion of preterm infants fail to respond to conventional inotrope treatment [5]. The reasons why preterm infants cannot maintain systemic blood flow and are not responsive to inotropes are unknown.

There is evidence suggesting that the profile of adrenoceptor subtypes in the heart changes with development [6]–[11], and it is acknowledged that the preterm infant may have a reduced number of β-adrenoceptors [2], [12]. However, evidence for this is contradictory. Two studies (in rabbits and mice) have investigated β-adrenoceptor abundance in the fetal period [8], [9], and both of these report reduced β-adrenoceptor abundance in the fetus at about 2/3 of gestation compared to term. In contrast, three studies of postnatal animals at a stage considered equivalent to the human preterm period, found no change in adrenoceptor number over this period, or greater numbers of β-adrenoceptors in the least mature animals [6], [11], [13]. All of these studies were conducted in animals that are very immature at birth where the heart is required to adapt to the *ex utero* circulation while still undergoing development (mice, rats and rabbits). There are no studies covering the preterm period in large animals with a similar degree of cardiac maturation at birth to that of the human infant. As adrenergic agonists such as dopamine and dobutamine continue to be a mainstay in the treatment of preterm infants with cardiovascular compromise, it is essential that we understand the profile of adrenoceptor expression prior to term. If there are fewer cardiac β-adrenoceptors in the preterm infant this could explain the failure of many preterm infants to respond to dopamine and dobutamine [5], and could also contribute to their poor cardiovascular function. In addition, if sexual dimorphism exists in expression, this could contribute to the greater mortality rates of preterm male infants and male lambs compared to females of the same species [14]–[16].

The levels and types of α-adrenoceptors expressed in the developing heart may also affect cardiac function and development. Increased expression of α_1A_-adrenoceptors enhances contractility in adult mice while over expression of the α_1B_ subtype induces left ventricular dysfunction [17]–[19]. α_2A_-adrenoceptors have been shown to influence cardiac growth and promote actin organization [20], [21]. Little is known about the expression profiles of α-adrenoceptor subtypes in the preterm heart.

Maternal glucocorticoid treatment is widely used to promote lung maturation of preterm infants. Glucocorticoid exposure reduces the incidence of low systemic flow [22] and promotes growth of the myocardium [23], [24]. Glucocorticoids have also been shown to increase cardiac expression of β-adrenoceptors in adult rats [25], [26], and to alter adrenoceptor expression in a variety of tissues in immature animals [27]–[29]. There are no data about the effect of glucocorticoid exposure on cardiac adrenoceptor expression in the preterm period. It is possible that the beneficial effects of glucocorticoids on preterm cardiovascular function [2], [22], [30] are mediated by alterations in cardiac adrenoceptor expression. If these effects are sex-specific, as many other effects of glucocorticoid exposure are [31], this could also contribute to the differing outcomes observed in male and female preterm infants.

We postulated that in the preterm heart β-adrenoceptor gene expression is low compared to the term heart, that mRNA abundance of α-adrenoceptor subtypes is altered compared to the term heart, and that antenatal glucocorticoid treatment would promote maturation of cardiac adrenoceptor expression. We tested these hypotheses by measuring the mRNA abundance of the subtypes of the α- and β-adrenoceptors, and by using saturation binding assays to measure the number of β-adrenoceptors on cardiac membranes isolated from male and female preterm and term piglets, and from male and female preterm piglets exposed to maternally administered glucocorticoids. β-adrenoceptor subtypes were investigated as these are the major target of the inotropes dopamine and dobutamine, commonly used in the neonate. α_1A_-adrenoceptors can also have inotropic effects [17], [32], whilst α_2A_- and α_2B_-adrenoceptors may influence cardiac growth and maturation [20]. The piglet was used because it has a similar pattern of cardiac maturation, both preterm and at term, to that of the human infant [33], [34].

## Materials and Methods

### Ethical Approval

The project was approved by The University of Queensland Animal Ethics Committee (AEC Approval Number: UQCCR/999/08) and conforms to the Australian Code of Practice for the Care and Use of Animals for Scientific Purposes (7th edition 2004). All surgery was performed under general anesthesia, and all efforts were made to minimize suffering.

### Animals

Large White X Landrace piglets were delivered by cesarean section at two ages, preterm piglets which were delivered at 91 days gestation (term = 115 d) and term piglets which were delivered two days before the expected farrowing date. At 91 days gestation age piglets are developmentally similar to approximately 23–25 weeks in a human infant. The piglet heart has a similar proportion of binucleated myocytes (a marker of maturity) to that in the human heart in both the preterm period (piglet 91d gestation and human at 23–28 weeks: 2–5%) and at term (piglet and human infant: about 10%) [33]–[35]. Cardiovascular function (blood pressure and need for cardiovascular support) in the piglet at 91d is similar to that in the human infant at 23–25 weeks [36]. This is the period where poor cardiovascular function and its treatment present the greatest difficulties to the neonatologist.

An additional group of preterm piglets (91 d) was exposed to maternally administered glucocorticoid (betamethasone, 0.19 mg/kg body weight, injected i.m.; Celestone Chronodose; Schering-Plough, USA) given 48 h and 24 h before delivery. The timing and dose/kg are equivalent to that given to women presenting with threatened preterm labour. In each preterm group (untreated preterm and preterm+glucocorticoid (GC)), 12 piglets (6 males, 6 females) from 3 litters/group were studied. For the term group, 11 piglets (5 males, 6 females) from 3 litters were studied. In most litters 2 male and 2 female piglets were studied, and at least one male and one female piglet were studied from every litter.

Details of cesarean section delivery have been published elsewhere [37]. Briefly, pregnant sows (280–350 kg) were premedicated with 400 mg azaperone given i.m. (Stresnil; Janssen, Australia). Anaesthesia was induced with 200 mg of alfaxalone given i.v. (Alfaxan-CD RTU; Jurox, Australia), followed by administration of additional alfaxalone as required to allow intubation of the trachea with a 14–16 mm endotracheal tube. The total administered dose of alfaxalone was 300–700 mg. Anaesthesia was maintained with 2% isoflurane (Attane Isoflurane USP; Minrad, USA) in O_2_ and sows breathed spontaneously. Throughout surgery, saline (2–3 L of 0.15 M NaCl) was administered via an ear vein and the following variables were monitored: arterial blood pressure by Doppler (Parks Medical Electronics Inc, Aloha, OR, USA), O_2_ saturation by pulse oximetry (Masimo, Irvine, CA, USA), end tidal isoflurane and end tidal CO_2_ concentrations (Capnomac Anaesthesia Monitor, Datex-Ohmeda Inc, Madison, WI, USA).

Caesarean delivery was performed via a ventral midline incision. Piglets were individually removed from the uterus at approximately 10min intervals. The piglet was anaesthetized with ∼5 mg/kg propofol via the umbilical vein (Provive 1%; AFT Pharmaceuticals, New Zealand). The umbilical cord was clamped and cut, and the piglet was weighed and sexed. The piglet’s chest was immediately opened and the heart was excised, weighed and dissected. The piglets had minimal exposure to anaesthetic and it is unlikely that this would have affected gene expression in the short time between exposure and tissue collection.

A small (∼0.5×0.5 cm) block of myocardium extending from epicardium to endocardium was obtained at the corner of the interventricular and atrioventricular grooves of each ventricular free wall. At the time of collection, the tissue block was snap frozen in liquid nitrogen and further crushed/chopped into small pieces for gene expression studies and receptor binding assays. After all piglets were delivered the sow was killed by i.v. injection of pentobarbital sodium (60ml Lethabarb, Virbac, Australia).

### Quantitative Real-time Reverse Transcription Polymerase Chain Reaction

Total mRNA was separated from approximately 50 mg of crushed tissue using Qiazol (Qiagen, Germany) according to the manufacturer’s instruction and purified using an RNeasy mini RNA extraction kit (Qiagen, Germany) with an on-column DNase I treatment using a DNase free RNase kit (Qiagen, Germany). An additional DNase I treatment using a Turbo DNase free kit (Life Technologies, Ambion), was carried out to minimize genomic DNA content. The integrity and concentration of each RNA sample was confirmed before and after the second DNase treatment using 1% agarose gel electrophoresis and absorbance spectrometry (NanoDrop1000, Thermo Fisher Scientific Inc.).

RNA was reverse transcribed using a High Capacity RNA-to-cDNA kit (Life Technologies, Applied Biosystems) according to the manufacturer’s guide. Real-time PCR was performed using commercial Taqman primers and probes (Life Technologies, Applied Biosystems). Target genes studied included the following adrenoceptor subtypes: β_1_- and β_2_-adrenoceptors (Gene symbol: *ADRB1*, product catalogue number: Ss03387549_s1; *ADRB2*, Ss03818941_s1), α_1A_-adrenoceptor (*ADRA1A*, Ss03387516_s1), α_2A_- α_2B_-adrenoceptors (*ADRA2A*, Ss03394899_s1; *ADRA2B*, Ss03379671_u1) and an endogenous control, glyceraldehyde-3-phosphate dehydrogenase (*GAPDH*, Ss03373286_u1, Applied Biosystems, USA). Each multiplex assay was subjected to a validation experiment to ensure that the efficiency of the target and endogenous control amplifications were approximately equal. Real-time PCR reactions were carried out on 10 ng cDNA with 180 nM forward and reverse primers and 50 nM probe for target genes, and 90 nM primers and 24 nM probe for *GAPDH* and Taqman Gene Expression Mastermix (Applied Biosystems). A Mastercycler ep *Realplex* ^2^ (Eppendorf South Pacific, Germany) was used. Data were analyzed using the comparative C_T_ (2^−ΔΔC^_T_) method as described previously [38]. Briefly, the mRNA level of each gene was normalized to GAPDH and expressed relative to term left ventricles (both sexes pooled). There was no effect of development or glucocorticoid exposure on *GAPDH* expression (*P*>0.3).

### Membrane Preparation and Radioligand Receptor Binding Assay for β-adrenoceptors

Tissues were prepared as previously described [39]. On the day of experiment, tissue blocks were suspended in lysis buffer (20mM sodium bicarbonate) and homogenized using an Ultra-Turax T125 with dispensing tool (N-18G; IKA Labotechnik, Staufen) 4 times for 10sec at 20500 rpm. Tissue homogenates were incubated on ice for 1h with occasional mixing by inversion. Large tissue debris was removed from the protein homogenate by low speed centrifugation (300×g for 1 min at 4°C). Membrane protein was then pelleted by high speed centrifugation (40000×g for 1 h at 4°C). The membrane pellet was resuspended in 1ml of incubation buffer (50 mM tris aminomethane, 1mM EGTA, 5mM MgCl_2_, pH 7.4). Protein concentration was determined by the Bradford method [40]. The membranes were further diluted in HEM buffer (20 mM HEPES, pH 7.4, 1.4 mM EGTA, and 12.5 mM MgCl_2_) to a uniform protein concentration of 0.5 μg/μl.

For all binding experiments, the total reaction volume was 350μl containing 100μg of membrane proteins in HEM buffer and the incubation time was 1h at room temperature. Reactions were terminated by vacuum filtration through Whatman GF/B filter paper using a Brandel 48-cell harvester (Brandel Inc.). Filters were transferred to scintillation vials, to which 4ml of Ultima-Gold Scintillation fluid (Beckman Coulter) was added. Reactivity was measured by liquid scintillation counting using a PerkinElmer liquid scintillation analyzer TriCarb-2800 TR (PerkinElmer, USA).

To determine the total number of β-adrenoceptor binding sites, saturation experiments were performed. Membranes suspended in HEM buffer were incubated with increasing concentrations of [^3^H]-dihydroalprenolol hydrochloride ([^3^H]-DHA, 0.5∼20 nM, β-adrenoceptor antagonist). Nonspecific binding was defined as binding in the presence of 50 μM propranolol. When sufficient tissue was available the relative contribution of each subtype to the total β-adrenoceptor binding sites was also determined using competition binding experiments. Membranes were incubated with 10nM [^3^H]-DHA and increasing concentrations (10^−12^∼10^−3 ^M) of the selective β_2_ antagonist ICI 118,551.

Nonlinear regression analysis of saturation and competition binding assay data was performed using an iterative curve fitting program Graphpad Prism (San Diego, CA, USA). Receptor binding site density (B_max_) for [^3^H]-DHA were calculated using the specific binding of the radioligand and converted into fmole per mg of protein. The competitive binding data for ICI 118,551 was tested for both one and two-site binding and the appropriate model was determined using the extra sum-of-squares F test. When two binding sites were present the ratio of high affinity to low affinity sites (β_2_:β_1_) was calculated.

### Data Analysis and Statistics

Data were analyzed with the statistical software program PASW Statistics 18 (version 18.0.0, SPSS Inc., Chicago, USA). To assess homogeneity of variance and normality of data, Levene and Shapiro-Wilk statistics were used. Data were transformed using logarithmic and square root functions where necessary. Effects of treatment group, sex and their interaction on receptor mRNA expression were detected using 3-way ANOVAs (with group and sex as fixed factors and litter as a random factor nested in group). Significant differences were reported only where these existed independently of litter effects. Where differences between piglet groups were detected, post hoc tests (Fisher’s least significant difference) were used to identify where these differences lay. Within each group, paired t-tests were used to compare between left and right ventricles. Effect of treatment group on total β-adrenoceptor binding density was detected using 3-way ANOVA (with group and sex as fixed factors and litter as a random factor nested in group). Effect of treatment group on β_1_-adrenoceptor binding density was detected using one-way ANOVA (with treatment group as a fixed factor). Statistical significance was set at *P*<0.05.

## Results

Cardiac adrenoceptor mRNA expression was assessed in 12 untreated preterm piglets (6 males, 6 females), 12 preterm piglets exposed to maternal glucocorticoid (GC) treatment (6 males, 6 females) and 11 term piglets (5 males, 6 females). Adrenoceptor subtypes (β_1_, β_2_, α_1A_, α_2A_ and α_2B_) were selected for their known effects on cardiac function and growth [2], [12], [17], [20], [32]. We were unable to determine expression of α_1B_-adrenoceptors because only a partially predicted mRNA sequence of *ADRA1B* for *sus scrofa* (pig) had been published and a reliable primer sequence was not available.

For all adrenoceptors studied, there was no difference in mRNA expression between male and female piglets in any group (LV and RV: *P*>0.1) and no interaction between sex and group (LV and RV: *P*>0.1). Therefore results from male and female piglets were combined.

### β-adrenoceptors

#### β_1_-adrenoceptor *(ADRB1)*

**Figure 1:**
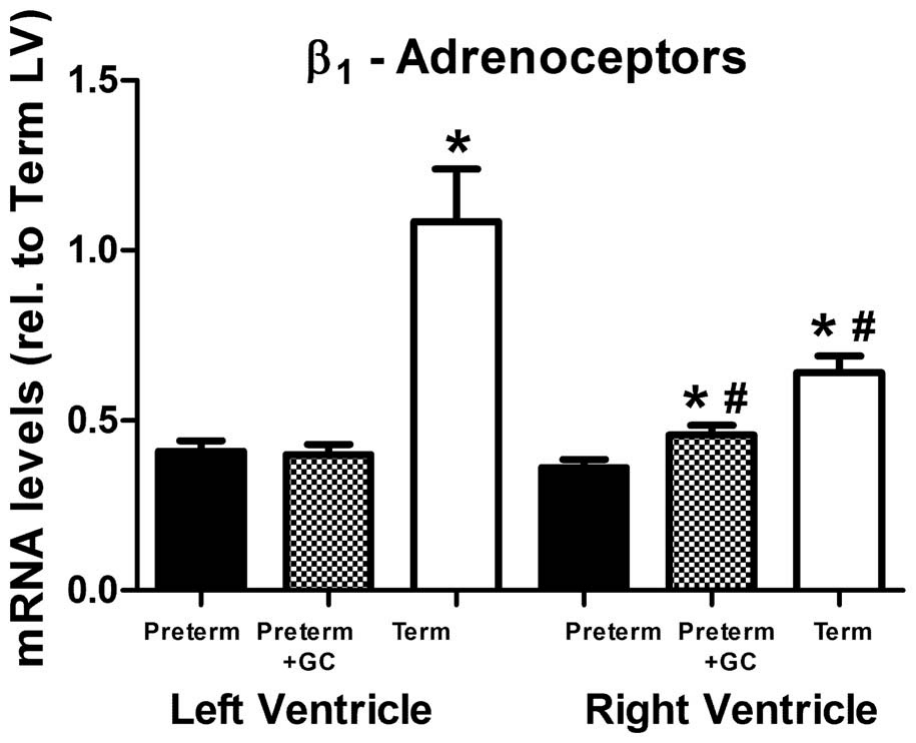
β_1_-adrenoceptor expression. β_1_-adrenoceptor (*ADRB1*) mRNA in left and right ventricle of untreated preterm (solid bar), preterm+GC (glucocorticoid exposed, dotted bar) and term (open bar) piglets. Mean ± SEM. N = 12 for all groups except term piglets where N = 11. * *P*<0.05 compared to untreated preterm group. **^#^** *P*<0.05 compared to left ventricle within the same group (paired t-test). Significant differences indicated only where these existed independently of litter effects.

*mRNA* levels in untreated preterm piglets were approximately 50% of that in term piglets in both left (LV) and right (RV) ventricles (Fig. 1, LV: *P*<0.001, RV: *P*<0.001). Following glucocorticoid treatment β_1_-adrenoceptor mRNA expression in the right ventricle was greater compared to untreated preterm piglets (Fig. 1, *P* = 0.008) but glucocorticoid treatment did not alter β_1_-adrenoceptor mRNA abundance in the left ventricle (*P*>0.1). At term, right ventricular mRNA abundances were less than in the left ventricle (Fig. 1, *P* = 0.004), however following glucocorticoid treatment in preterm piglets, expression in the right ventricle was greater than the left ventricle (*P* = 0.014).

#### β_2_-adrenoceptor *(ADRB2)*

**Figure 2:**
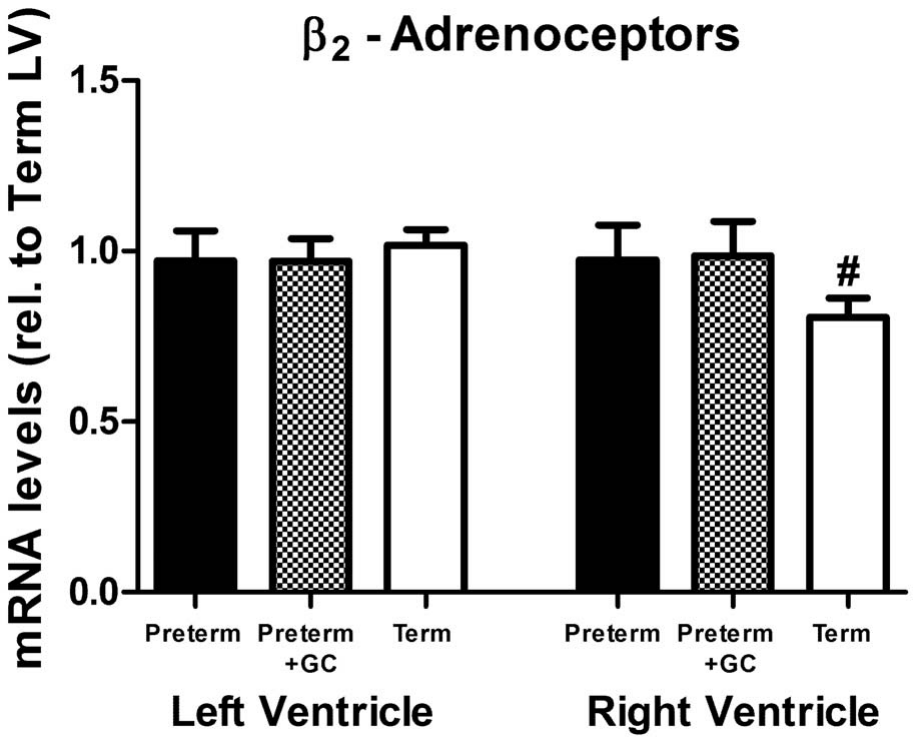
β_2_-adrenoceptor expression. β_2_-adrenoceptor (*ADRB2*) mRNA in left and right ventricle of untreated preterm (solid bar), preterm+GC (glucocorticoid exposed, dotted bar) and term (open bar) piglets. Mean ± SEM. N = 12 for all groups except term piglets where N = 11. ^#^ P<0.05 compared to left ventricle within the same group (paired t-test). Significant differences indicated only where these existed independently of litter effects.

*mRNA* levels were the same in term and untreated preterm piglets and were not altered in glucocorticoid exposed animals (Fig. 2, LV and RV: *P*>0.1). At term right ventricular β_2_-adrenoceptor mRNA abundances were less than in the left ventricle (Fig. 2, *P* = 0.002).

### α-adrenoceptors

#### α_1A_-adrenoceptor *(ADRA1A)*

**Figure 3:**
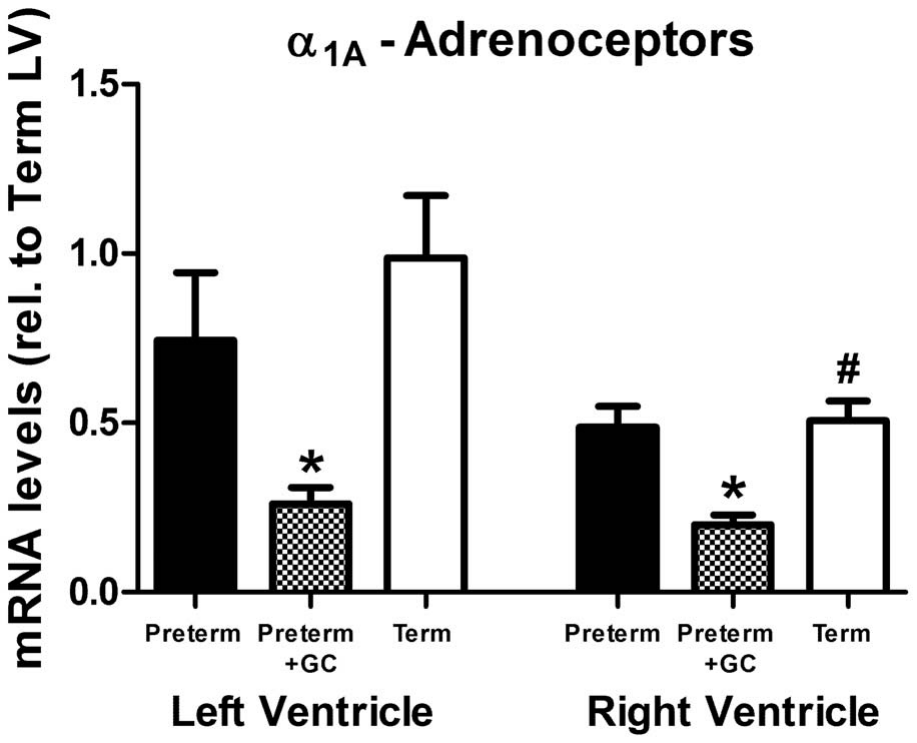
α_1A_-adrenoceptor expression. α_1A_-adrenoceptor (*ADRA1A*) mRNA in left and right ventricle of untreated preterm (solid bar), preterm+GC (glucocorticoid exposed, dotted bar) and term (open bar) piglets. Mean ± SEM. N = 12 for all groups except term piglets where N = 11. * *P*<0.05 compared to untreated preterm group. ^#^ *P*<0.05 compared to left ventricle within the same group (paired t-test). Significant differences indicated only where these existed independently of litter effects.

*mRNA* levels in untreated preterm piglet hearts were the same as in term piglets (Fig. 3, LV: *P* = >0.1, excluding one outlier >2 SD above mean, RV: *P*>0.1), but in piglets exposed to maternal glucocorticoid treatment α_1A_-adrenoceptor mRNA abundance was less in both left and right ventricles compared with mRNA abundance in untreated preterm piglets (Fig. 3 LV: *P* = 0.008, RV: *P*<0.001). At term, the relative abundance of α_1A_-adrenoceptor mRNA in the right ventricle was less than that in the left ventricle (Fig. 3, *P* = 0.008).

#### α_2A_-adrenoceptor *(ADRA2A)*

**Figure 4:**
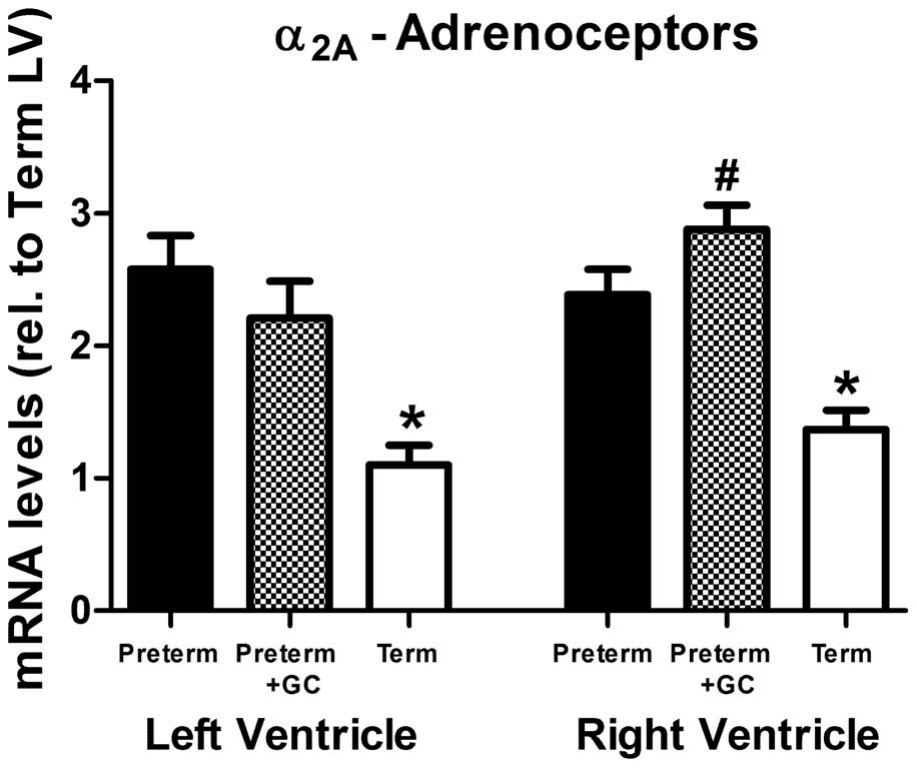
α_2A_-adrenoceptor expression. α_2A_-adrenoceptor (*ADRA2A*) mRNA in left and right ventricle of untreated preterm (solid bar), preterm+GC (glucocorticoid exposed, dotted bar) and term (open bar) piglets. Mean ± SEM. N = 12 for all groups except term piglets where N = 11. * *P*<0.05 compared to untreated preterm group. ^#^ *P*<0.05 compared to left ventricle within the same group (paired t-test). Significant differences indicated only where these existed independently of litter effects.

*mRNA* abundance in both left and right ventricles of untreated preterm piglets was greater than at term (Fig. 4, LV: *P*<0.001, RV: *P* = 0.001). Expression in glucocorticoid exposed preterm piglets was not different to untreated preterm piglets (Fig. 4, LV: *P*>0.1, RV: *P* = 0.059). In glucocorticoid exposed preterm piglets, expression in the right ventricle was greater than in the left ventricle (*P* = 0.028).

#### α_2B_-adrenoceptor *(ADRA2B)*

*mRNA* was not detected in any heart.

### β-adrenoceptor Binding Site Density

As β-adrenoceptors are the major cardiac target for drugs that are currently in clinical use as support for preterm cardiovascular function, we investigated if the changes in gene expression described above resulted in an increased number of functional cardiac β-adrenoceptors receptors. Insufficient tissue was available to also determine binding site density of α-adrenoceptors.

**Figure 5:**
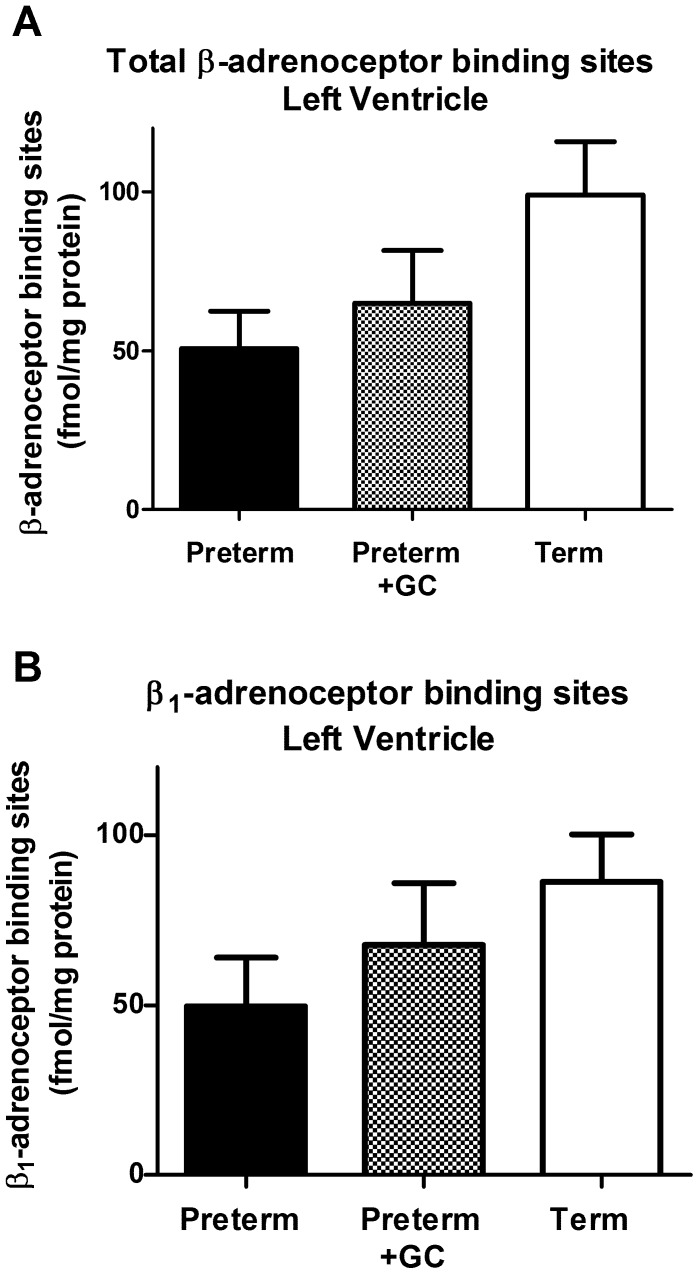
β-adrenoceptor binding site density. Total β-adrenoceptor binding site density (Panel A) and β_1_-adrenoceptor binding site density (Panel B) in left ventricle in untreated preterm (solid bar), preterm+GC (dotted bar) and term piglet hearts (open bar). Receptor density was determined from saturation binding assay using the β-adrenoceptor antagonist [^3^H]-dihydroalprenolol hydrochloride. β_1_-adrenoceptor binding site density was calculated from the B_max_ (fmol/mg) obtained from the saturation binding assay x fraction low affinity binding sites obtained from the competition binding assay using ICI 118,551 (β_2_ selective antagonist). Data are mean ± SEM. For total β-adrenoceptor binding site density N = 11–12 for all groups. For β_1_-adrenoceptor binding site density N = 9–11 for all groups.

The mean total β-adrenoceptor binding site density in the left ventricle was less in the preterm group compared to term, although this did not quite reach significance (Fig. 5A, *P* = 0.070 for group effect). There was no difference between male and female piglets (*P*>0.1) and no interaction between group and sex (*P*>0.1).

Most of the β-adrenoceptor binding sites in the LV were β_1_-adrenoceptor binding sites. In 31 piglets ICI 118,551 binding displayed a single lower affinity binding site indicating that only β_1_-adrenoceptor binding sites were present. In four piglets out of the total of 35 piglets across all three groups β_2_-adrenoceptor binding sites were detected in the left ventricle. This included 3 term piglets (one male, two females) and one untreated preterm male piglet. The fraction of β_2_-adrenoceptor binding sites in the left ventricle was 12% in the preterm piglet and ranged from 22–60% of total β-adrenoceptor binding sites in the term piglets (5.2–86.2 fmol/mg of membrane protein).

Data for β_1_-adrenoceptor binding showed the same trend as for total β-adrenoceptors (Fig. 5B) although results did not reach statistical significance (*P* = 0.169).

## Discussion

This study of adrenoceptor expression in the preterm piglet heart is the first to investigate a range of adrenoceptor sub-types in a species with a pattern of cardiac maturation that is similar to the human infant [33], [34], [41], and the first to separately assess right and left ventricle, male and female animals, and the effects of maternal glucocorticoid treatment. The study produced a number of significant results.

First, we found that β_1_-adrenoceptor mRNA abundance was lower in the ventricular myocardium of untreated premature piglets compared with term piglets (Fig. 1). This was the case in both left and right ventricle. The density of [^3^H]-DHA binding sites showed a similar pattern, although the differences were not statistically significant, possibly because of the greater variability in these measures and the smaller amount of data available (Fig. 5).

There are very few previous studies that specifically focus on β_1_-adrenoceptors in the preterm heart and these studies do not distinguish between left and right ventricles nor do they address the possibility of sexual dimorphism or investigate the effects of glucocorticoids. They do show in rabbits [8] and mice [9], that the density of cardiac β-adrenoceptor binding sites is lower in preterm hearts compared to term hearts. In contrast, studies in the postnatal rat, at a stage considered equivalent to the preterm period, found no change in receptor numbers over this period, or even greater numbers of β-adrenoceptors in the least mature animals [6], [11], [13]. Our findings in the piglet, are similar those in fetal rabbits [8] and mice [9].

A lower density of cardiac β_1_-adrenoceptors in the preterm human infant could limit the response of the preterm heart to endogenous catecholamines and contribute to the development of cardiovascular insufficiency that often occurs in the human preterm. A lower density of cardiac β_1_-adrenoceptors could also explain the lack of response by many preterm babies to exogenous inotrope treatments such as dopamine and dobutamine [5]. Despite evidence of reduced β-adrenoceptor density in the preterm heart and the significant treatment failure rate, dopamine and dobutamine are effective in some infants and continue to be the mainstay of treatment for cardiovascular compromise in the preterm infant. Is it likely that variability exists within the human population just as we have seen in the piglet, particularly in receptor binding studies, so that some babies do not have a critical mass of receptors required for an effective response while others do. There is a need to understand the factors responsible for the developmental increase in expression in the piglet and other species [8], [9], as this may contribute to the improved response of human infants to inotropes 48h after birth, and could provide a suitable target for improving the efficacy of current treatments. It is also important to examine expression and function of adrenoceptors in peripheral blood vessels. If the adrenoceptor profile of peripheral blood vessels is different in the preterm neonate, the response to both endogenous catecholamines and to dopamine and dobutamine may not be as expected based on responses in adults. This difference might also contribute to poor cardiovascular function, and to the poor response to inotropic support with excessive vasoconstriction and a reduction in cardiac output as has been observed in some babies [42].

Second, this study showed that there were no differences in cardiac β_1_-adrenoceptor mRNA abundance between male and female piglets. This suggests that the increased mortality and morbidity observed in male preterm infants compared to females [14]–[16] is probably not the result of differences in β_1_-adrenoceptor gene expression.

The expression of β_1_-adrenoceptors in the preterm left ventricle was also unaffected by maternal glucocorticoid treatment. In contrast expression was increased in the right ventricle. Glucocorticoid exposure also has ventricle specific effects on ventricular weight with increases in left but not right ventricular weight, although this only occurred in female piglets [34]. It is not clear why expression is altered only in one ventricle and whether upregulation in the right but not the left ventricle would contribute to the reduced need for circulatory support observed in preterm infants after maternal glucocorticoid treatment [2], [22], [30].

This is not the first report of an association between glucocorticoid exposure and altered β-adrenoceptor expression. Glucocorticoids increase β_1_-adrenoceptor mRNA levels and the number of receptor binding sites in the adult rat heart [25], [26]. A transient increase in receptor expression was also seen in the liver of porcine fetuses in response to maternally administered dexamethasone [43]. This study does provide the first evidence that antenatal maternal administration of glucocorticoids (using a regimen and dosage similar to that used clinically) affects fetal cardiac β-adrenoceptor expression. This may partially explain the improvement in cardiac function following antenatal glucocorticoid treatment [2], [30].

The third important finding of this study is that α_1A_-adrenoceptor mRNA abundance in the left and right ventricles is the same in preterm and term piglets but reduced in preterm piglets exposed to maternal glucocorticoid treatment (Fig. 3). In the adult rat heart a transient increase in the number of α_1_-adrenoceptor binding sites was seen after glucocorticoid treatment [25], however this could have been due to increases in α_1B_- and α_1D_-adrenoceptor expression rather than α_1A_-adrenoceptors [44]. It is possible that α_1A_-adrenoceptors, as well as β_1_-adrenoceptors, have inotropic effects. Cardiac muscle targeted overexpression of α_1A_-adrenoceptors in adult mice increased myocardial contractility [17], [32]. There is also evidence that in pathological conditions when the cardiac β-receptor signaling pathway is attenuated, α_1_-receptors take over their inotropic role [19], [45]. However, in our study α_1_-receptor mRNA abundance in preterm piglets was not increased relative to term. In addition glucocorticoid exposed preterm hearts may be disadvantaged by having fewer α_1A_-adrenoceptors. The observation that mRNA abundance of α_1A_-adrenoceptors in the preterm heart is the same as that at term suggests that treatments that target α_1A_-adrenoceptors may be effective for supporting preterm cardiac function, although an alternative treatment would be required when antenatal glucocorticoids are administered because these receptors are severely suppressed.

The level of α_2A_-adrenoceptor mRNA abundance was greater in untreated preterm hearts than in term animals (Fig. 4). This is consistent with observations in the rat showing that the number of α_2_-adrenoceptors in the heart falls during the first week of life [46]. The increased level of expression in the preterm heart may simply reflect the stage of cardiac development where the preterm heart is growing rapidly and myofilaments are becoming more organized [47]. In fetal rats adrenoceptor agonists enhanced actin cytoskeleton organization in cardiac myocytes and these effects were blocked by α_2_-antagonists [20]. The lack of expression of α_2B_-adrenoceptor in preterm and term hearts in piglets is in accordance with findings in other species [20].

A limitation of the study was the inability to unequivocally show that changes in adrenoceptor mRNA abundance translated to increased levels of receptor protein and/or receptor binding density. It was not possible to perform western blotting to measure adrenoceptor protein levels because the adrenoceptor antibodies are not specific to receptor subtype [48], [49]. Although ligand binding studies for left ventricular β-adrenoceptors showed changes that were similar to the gestational changes in mRNA abundances, this did not quite reach statistical significance. As noted by previous authors, ligand binding studies are “laborious and difficult when cell or tissue amounts are limiting” as was the case in the current study, and we were not able to access tissue from additional animals to increase the statistical power for binding studies. We were also unable to measure α-adrenoceptor binding density because of lack of sufficient amounts of cardiac tissue, so we chose to focus on left ventricular β-adrenoceptor binding density because these receptors are the most clinically relevant when considering the efficacy of currently used inotropes.

In conclusion, the low level of expression of β_1_-adrenoceptors in the preterm heart, previously examined only in species born very immature, has been confirmed by mRNA abundance in an animal with a similar cardiac maturation profile to the human infant. Reduced expression of β_1_-adrenoceptors may contribute to the poor cardiovascular function seen in preterm infants, and result in failure of the preterm infant to respond to inotropes commonly used to treat poor cardiovascular function. Further study is needed to confirm the functional significance of this observation. Antenatal glucocorticoid exposure does not increase expression of β_1_-adrenoceptors in the left ventricle. There is reduced mRNA abundance of α_1A_-adrenoceptors in glucocorticoid exposed preterm hearts which may exacerbate any difficulties associated with low levels of β_1_-adrenoceptor expression. The difference in mortality and morbidity of male and female neonates is not explained by differences in cardiac adrenoceptor expression.
